# Total and Differential Phylloquinone (Vitamin K_1_) Intakes of Preterm Infants from All Sources during the Neonatal Period

**DOI:** 10.3390/nu7105393

**Published:** 2015-09-25

**Authors:** Paul Clarke, Simon J. Mitchell, Martin J. Shearer

**Affiliations:** 1 Neonatal Unit, Norfolk and Norwich University Hospitals NHS Foundation Trust, Colney Lane, Norwich, Norfolk NR4 7UY, UK; 2 Newborn Intensive Care, St Mary’s Hospital, Oxford Road, Manchester M13 9WL, UK; simon.mitchell@cmft.nhs.uk; 3 The Centre for Haemostasis and Thrombosis, St. Thomas’ Hospital, Westminster Bridge Road, London SE1 7EH, UK; martin.shearer@gstt.nhs.uk

**Keywords:** phylloquinone, prophylaxis, prematurity, deficiency, bleeding, dietary, micronutrients, supplement, nutrition

## Abstract

All newborns require phylloquinone after birth to prevent vitamin K deficiency bleeding. Babies born prematurely may be at particular risk of deficiency without adequate supplementation during infancy. The main sources of phylloquinone in preterm babies during the neonatal period are the prophylactic dose of phylloquinone given at birth, and that derived from parenteral and/or enteral feeding. This observational study formed part of a prospective, multicentre, randomised, controlled trial that examined the vitamin K status of preterm infants after random allocation to one of three phylloquinone prophylactic regimens at birth (0.5 or 0.2 mg intramuscularly or 0.2 mg intravenously). In this nutritional sub-study we quantified the proportional and total phylloquinone intakes of preterm infants within the neonatal period from all sources. Almost all infants had average daily phylloquinone intakes that were in excess of the currently recommended amounts. In infants who did not receive parenteral nutrition, the bolus dose of phylloquinone given at birth was the major source of phylloquinone intake, whereas in infants who received parenteral nutrition, the intake from the parenteral preparation exceeded that from the bolus dose by a ratio of approximately 3:1. Our study supports the concern of others that preterm infants who receive current parenteral nutrition formulations may be receiving excessive vitamin K.

## 1. Introduction

Vitamin K-dependent coagulation factors are synthesised exclusively in the liver and so the maintenance of adequate hepatic vitamin K reserves is essential for normal haemostasis. Preterm as well as term babies are born with extremely low hepatic stores of vitamin K [1,2]. Unlike in adults, the major hepatic form of vitamin K in neonates is phylloquinone (vitamin K_1_) which is also the major form of the vitamin in human breast milk. After birth, the combination of low hepatic reserves and relatively low concentrations of phylloquinone in breast milk (compared to formula milks) places the breast-fed infant at increased risk of developing vitamin K deficiency. They are consequently dependent upon adequate intakes of vitamin K postnatally and during early infancy to keep them healthy. Supplementary vitamin K prophylaxis is offered at birth to protect against vitamin K deficiency bleeding (VKDB), a now rare but still potentially lethal disorder which may cause devastating brain injury and lifelong impairment in survivors [3,4,5]. Preterm infants may be at higher risk of developing VKDB without adequate ongoing phylloquinone intakes following birth [6], so a baseline understanding of their current typical intakes from various sources during the neonatal period is clearly important.

In both term and preterm infants the very large bolus dose of phylloquinone given prophylactically at birth is considered to be the major source of phylloquinone in early infancy [7,8], and, therefore, to provide the mainstay of protection provided against VKDB during early infancy. Preterm neonates receive an on-going supply of phylloquinone from dietary sources, including from enteral and often parenteral feeding. No previous study has reported in detail the total phylloquinone intakes of preterm infants from all the various sources during their first weeks of life. The aims of this study were: (i) to quantify phylloquinone intake by preterm infants from all sources during the first postnatal weeks of life; and (ii) to assess the relative contribution of the various sources to the total phylloquinone intake of preterm infants during the neonatal period.

## 2. Experimental Section

### 2.1. Study Design

This observational study was done as part of a prospective, multicentre, randomised controlled trial that examined the vitamin K status of preterm infants after random allocation to one of three phylloquinone prophylactic regimens at birth [9]. The setting was three neonatal intensive care units (NICUs) in the United Kingdom (UK). The present study reports the detailed phylloquinone intakes of these infants during the first weeks after birth. Data on the functional outcome measures of phylloquinone status and metabolism in this cohort, including undercarboxylated prothrombin (PIVKA-II), prothrombin time, and factor II concentrations, were reported previously [9]. Assessment of other vitamin-K dependent proteins (such as osteocalcin and matrix Gla protein) was beyond the scope of the study.

### 2.2. Subjects

Infants eligible for inclusion were born at a gestational age of <32 completed weeks and admitted to one of the three participating NICUs. Exclusion criteria included fetal intracranial haemorrhage suspected antenatally, history of maternal antiplatelet antibodies or alloimmune thrombocytopenia, maternal drug treatment with known vitamin K antagonists, life-threatening congenital abnormality, and marked bruising present from birth. Infants with bowel diseases (such as necrotising enterocolitis) or other expected complications of prematurity were not excluded or discontinued from the study. Written parental consent was obtained for inclusion, and the period of recruitment was between November 2001 and February 2003.

### 2.3. Sources of Vitamin K Intake

There are only three prospective sources of phylloquinone intake by preterm infants during the neonatal period: (i) the prophylactic bolus dose received soon after birth and any extra bolus doses given while in the NICU; (ii) enteral milk feeds and human milk fortifiers (HMFs); and (iii) parenteral nutrition (PN).

#### 2.3.1. Prophylactic Phylloquinone Formulation and Dose

The phylloquinone preparation provided for prophylaxis and for any subsequent bolus dose for infants in this study was Konakion^®^ Neonatal Ampoules (Roche Ltd, Basel, Switzerland). This formulation contained phytomenadione 2 mg/mL and had Cremophor EL (BASF AG, Ludwigshafen, Germany) as the solubilising excipient. Although unlicensed for use in preterm infants, this vitamin K formulation was at the time the commonest preparation used for prophylaxis in the UK [10].

For the main study [2,9], infants were randomly allocated to receive one of three vitamin K prophylaxis regimens within a few hours of birth: phylloquinone 0.5 mg intramuscularly (IM), 0.2 mg IM, or 0.2 mg intravenously (IV). As a safety provision, the study protocol provided that a 0.2 mg IM additional bolus dose of phylloquinone would be given during the study period to any infant with abnormal coagulation values or clinical bleeding in case these findings represented vitamin K deficiency.

#### 2.3.2. Enteral Feeding and Human Milk Fortifiers

The introduction and grading of enteral feeds proceeded in line with standard practices. In general, small “trophic” volumes (totalling ~20 mL/kg/day) of colostrum/human milk and/or a preterm milk formula were commenced within the first few days postnatal. Maternal expression of breast milk was actively encouraged, and expressed breast milk (EBM) was the preferred milk feed for preterm infants at all participating neonatal units. Bolus aliquots of milk were given via a nasogastric tube, initially on an hourly basis. Any increment in the feed volume was a decision of the clinical ward round and would depend upon an infant’s stability and any recent history of feed tolerance. Infants were considered to have established full enteral feeds when they were tolerating a volume of at least 150 mL/kg/day of milk and no longer on PN. An infant who tolerated the gradual feed increase without event or setback would potentially establish full feeds by 7–10 days postnatal.

It was standard clinical practice at all three participating NICUs to add HMF routinely to breast milk once the full enteral feed volume of 150 mL/kg/day was attained. Three commercially-available HMFs were available in the UK during the study period. Infants at the participating units all received Eoprotin HMF (Nutricia Ltd., Trowbridge, UK). Coincidentally, a new formulation of Eoprotin with a higher phylloquinone content was introduced nationally in the UK during the study period. Initially one scoop was given per 100 mL of breast milk, and this was graded up to three scoops (old Eoprotin formulation) or four scoops (new Eoprotin formulation) on successive days as tolerated. Infants discharged to neonatal centres outside of the three trial centres prior to study completion who subsequently received a different HMF had their phylloquinone intake calculated according to the actual fortifier received.

#### 2.3.3. Parenteral Nutrition

Administration of PN solution was routine for the sickest neonates in the participating NICUs. It was generally reserved for all very preterm infants (less than ~29 weeks’ gestation), extremely low birth weight infants (ELBW; <1000 g), and also any other neonate that was considered unlikely to achieve full enteral feeding by 7–10 days postnatal. It was expected that many of the more mature and heavier preterm infants in the study group would not require PN.

PN was usually commenced within the first three days of life. The daily volume of lipid infused was routinely graded up over the first three days to minimise fat intolerance and hyperlipidaemia. The phylloquinone-containing components of the PN solution were 20% Intralipid^®^, an intravenous fat emulsion, and Vitlipid N Infant^®^, a fat-soluble vitamin emulsion (Fresenius Kabi Ltd, Runcorn, Cheshire, UK). Nursing charts recorded the dates when PN began and ended, and also the exact daily volumes of the respective PN solutions delivered to each infant.

### 2.4. Assessment of Phylloquinone Intake

Phylloquinone intakes from all sources were calculated between birth and the date that the infant had tolerated full enteral feeds (*i.e.*, ≥150 mL/kg/day of milk) for a continuous full 2-week period, the latter date being considered the date of “study completion”. The total vitamin intake was thus calculated for each enrolled infant between birth and the date that full enteral feeds had been tolerated for two weeks.

Each infant’s absolute phylloquinone intake between birth and study completion was calculated from the sum amounts received from: (i) allocated prophylactic phylloquinone dose and any extra bolus phylloquinone doses given during the study period, obtained from review of the prescription charts; (ii) daily enteral feed volumes and HMF, obtained from the daily nursing charts; and (iii) PN intake, with daily volumes of Intralipid and Vitlipid received being obtained from the PN prescription charts and the nursing infusion administration charts.

An average daily phylloquinone intake was calculated for each infant from its total phylloquinone intake (expressed in μg/kg, using the infant’s weight at completion of the study) divided by the number of days from birth to study completion. The phylloquinone intake from enteral feeds was calculated from the actual milk types and volumes fed to each infant between birth and study completion, as recorded on the neonatal nursing charts.

#### 2.4.1. Phylloquinone Content of Milk Feeds and Fortifier

Table 1 shows the phylloquinone content of human preterm breast milk, and of the preterm artificial milk formulae that were available in the UK during the study period. The values for phylloquinone content of term infant human breast milk and term infant milk formulae are also provided for comparison purposes. Milk formulae phylloquinone contents are compiled from the various manufacturers’ contemporaneous data sheets. Values for the phylloquinone content of HMF-fortified preterm breast milk are also provided; these are based upon the manufacturer-published phylloquinone content of the various HMFs that were commercially-available during the study period, and upon the mean phylloquinone concentration value reported for preterm EBM. The value for mean phylloquinone concentration of human preterm EBM (3.0 μg/L) derives from a study of human milk obtained on postnatal one day from mothers of preterm infants prior to maternal phylloquinone supplementation [11]. To date this study of Bolisetty *et al*. [11] remains the only known study in which breast milk phylloquinone concentrations have been measured in mothers of preterm infants. Nevertheless, this mean value compares well to the mean concentration of phylloquinone in mature human breast milk (2.1 μg/L) from mothers of term infants reported by Haroon *et al.* [12], and to the mean value (2.5 μg/L) that was used in the calculation of the Adequate Intake (AI) by the Food and Nutrition Board [7]. Addition of HMF to EBM provided a total phylloquinone concentration of 5–66 μg/L depending on brand used (Table 1). Artificial preterm milk formulae provided 40–66 μg/L of phylloquinone depending on brand. These values were used to calculate exact phylloquinone intakes from the enteral feeds for all study individuals up to completion of the study.

**Table 1:** Phylloquinone content of human milk, commercial milk formulae, and fortifiers.

Nutrient	Phylloquinone Content (μg/100 mL)	Phylloquinone Content (μg/L)
Human Milk		
Term colostrum ^a^	0.23	2.3
Term mature EBM ^a^	0.21	2.1
Preterm colostrum/EBM ^b^	0.30	3.0
Formula Milks		
PreAptamil	6.6	66
Aptamil	4.0	40
Nutriprem 1	6.6	66
Nutriprem 2	5.9	59
SMA Low Birth Weight	8.0	80
SMA Gold	6.7	67
C + G Premium	5.1	51
C + G Plus	5.0	50
Human Milk Fortifiers		
Eoprotin (old formulation)	0.2 per 3 scoops (3 g powder) ^c^	2
Eoprotin (new formulation)	6.3 per 4 scoops (4.2 g powder) ^c^	63
Nutriprem HMF	6.2 per 2 sachets (4.2 g powder) ^c^	62
SMA Breast Milk Fortifier	11.0 per 2 sachets (4 g powder) ^c^	110
Fortified EBM ^d^		
Eoprotin old formulation	0.5	5
Eoprotin new formulation	6.6	66
Nutriprem HMF	6.5	65
SMA HMF	11.3	113

EBM, expressed breast milk; HMF, human milk fortifier. ^a^ Analysis of colostrum and breast milk obtained within five days *post-partum*, from Haroon *et al.* [12]; ^b^ Mean preterm EBM concentration on the first postnatal day in unsupplemented mothers, from Bolisetty *et al.* [11]; ^c^ Quantities recommended by manufacturers for addition to each 100 mL of human milk; ^d^ Values listed refer to content of fortified human milk after adding the recommended amount of HMF according to manufacturers’ instructions

#### 2.4.2. Phylloquinone Content of Parenteral Nutrition Solution

The phylloquinone content of Vitlipid N Infant (fat soluble vitamin emulsion) was 20 mg/L. That of 20% Intralipid IV fat emulsion varied between batches, and ranged from 0.50 to 0.77 mg/L [13]; for calculation purposes a figure of 0.6 mg/L was used. Infants on PN received 4 mL/kg of Vitlipid and 5–15 mL/kg of 20% Intralipid per day, providing daily phylloquinone supplementation of 80 μg/kg and 3–9 μg/kg respectively.

#### 2.4.3. Calculation of Total and Proportional Phylloquinone Intake from All Sources

The average daily phylloquinone intake was calculated for each infant from the total phylloquinone intake (expressed as μg/kg body weight at study completion) divided by number of days between birth and study completion. The proportional intake of phylloquinone from each route was also assessed: for each infant the contribution to overall total intake from each phylloquinone source was expressed as a percentage of total intake. In addition to calculating the proportional intake for all infants who completed the study (because not all preterm infants required or received PN feeding) the proportional intake was calculated separately for infants who received PN and for infants who did not receive any PN.

#### 2.4.4. Statistical Analysis

Infants who died before study completion were excluded from analysis. Data for all randomised infants who completed the study were analysed using StatsDirect statistical software (www.statsdirect.com) version 2.4.5. The Mann-Whitney U test was used to compare phylloquinone intakes between the three randomisation cohorts, and a *p*-value of <0.05 was considered statistically significant.

### 2.5. Ethics Approvals

The study received prior ethical review board approval from Salford and Trafford Local Research Ethics Committee on 5 November 2001 (Project No. 01198). Further Research Ethics Committee approvals were given on 10 July 2002 and on 10 December 2002 for extension of the study to two additional sites.

## 3. Results

### 3.1. Baseline Characteristics

Of 98 infants randomised to the main trial [2,9], 15 died before study completion and three infants with incomplete data were excluded from the analysis. All deaths were related to the common complications of prematurity and no infant in the study had vitamin K deficiency bleeding [9]. A total of 80 infants completed the study. Table 2 shows their baseline characteristics, including feeding characteristics, overall and subgrouped according to initial allocated dose of phylloquinone prophylaxis. There were no significant differences between subgroups for any of these baseline characteristics. As already reported, all infants had satisfactory vitamin K status at study completion and there were no significant differences in any functional outcome measures of phylloquinone status and metabolism between the subgroups in this cohort [9]. No infant suffered any suspected adverse event or reaction in relation to phylloquinone dosage or its route of administration. There were no differences between sub-groups in incidence of significant intraventricular haemorrhage (*n* = 6 infants overall, *p* = 0.9), other significant bleeding, such as gastro-intestinal or pulmonary (*n* = 7 infants overall, *p* = 0.2), or numbers transfused with fresh frozen plasma during the study (*n* = 7 infants overall, *p* = 0.7).

**Table 2:** Baseline characteristics of the *n* = 80 infants who completed the study and subgroups according to initial phylloquinone prophylaxis regimen.

Characteristic	All Infants (*n* = 80)	0.5 mg IM (*n* = 28)	0.2 mg IM (*n* = 26)	0.2 mg IV (*n* = 26)
Gestational Age, weeks	29.4 (22.4–31.9)	29.0 (24.1–31.9)	29.9 (24.0–31.7)	29.6 (22.4–31.4)
Birth Weight, g	1092 (454–1910)	1033 (454–1910)	1143 (534–1910)	1203 (575–1892)
Male gender, *n*	36 (45%)	10 (36%)	13 (50%)	13 (50%)
Received any PN, *n*	56 (70%)	18 (64%)	17 (65%)	21 (81%)
Duration of PN ^a^, days median (range) (IQR)	10 (3–78) (7–17)	9 (4–78) (7–13)	10 (5–29) (9–21)	9 (3–48) (5–18)
Postnatal age reached full enteral feeds, days	10 (4–80)	9 (4–80)	12 (4–43)	11 (4–49)
Postnatal age at study completion, days	25 (17–90)	24 (19–90)	26 (19–58)	26 (17–64)
Weight at study completion, g	1495 (600–2710)	1281 (605–2650)	1595 (600–2270)	1572 (612–2710)

Data are median (range) unless indicated. IM, intramuscular; IV, intravenous; PN, parenteral nutrition; IQR, inter-quartile range. ^a^ Data shown here include only babies who received any period of PN. There were no statistically significant differences between any subgroups for any of the baseline characteristics shown.

### 3.2. Total Phylloquinone Intakes

Table 3 shows total phylloquinone intakes from all sources between birth and study completion overall and for the subgroups. There were no significant differences in absolute phylloquinone intakes from all sources between the three study groups at study completion. However, after correcting for birth weight and days to study completion, the average daily intake of phylloquinone adjusted for birth weight was significantly lower for infants who had received 0.2 mg boluses at birth (*p* < 0.05).

**Table 3:** Phylloquinone intakes from all sources between birth and study completion (adapted from Clarke *et al.* [9], with permission).

Phylloquinone Intake (µg)	All infants (*n* = 80)	0.5 mg IM (*n* = 28)	0.2 mg IM (*n* = 26)	0.2 mg IV (*n* = 26)
***Via bolus doses***				
Mean (SD)	378 (256)	629 (264)	238 (80) *	246 (130) *
Median (range)	200 (200–1500)	500 (500–1500)	200 (200–400) *	200 (200–800)
***From PN*** ^†^				
Mean (SD)	784 (939)	610 (972)	754 (768)	1001 (1045)
Median (range)	542 (0–5071)	440 (0–5071)	674 (0–2441)	713 (0–3895)
***Via enteral feeds***				
Mean (SD)	124 (90)	118 (85)	125 (97)	131 (91)
Median (range)	133 (2–340)	116 (2–340)	122 (6–304)	145 (8–300)
***Total phylloquinone intake µg***				
Mean (SD)	1286 (1025)	1357 (1133)	1118 (775)	1378 (1136)
Median (range)	1011 (214–6574)	1038 (507–6574)	958 (332–2730)	1020 (214–4822)
***µg/kg***				
Mean (SD)	914 (597)	1070 (633)	808 (591)	853 (548)
Median (range)	775 (141–2594)	1048 (408–2594)	699 (173–2057)	704 (141–2343)
***µg/kg/day***				
Mean (SD)	30 (15)	37 (15)	26 (15) ^‡^	27 (11) ^‡^
Median (range)	28 (7–75)	39 (18–75)	27 (8–55) ^‡^	27 (7–65) ^‡^

SD, standard deviation. ^†^ Includes infants who received any period of PN as well as those who did not receive PN; * *Versus* 0.5 mg IM group, *p* < 0.001; ^‡^ *Versus* 0.5 mg IM group, *p* < 0.05.

#### 3.2.1. Phylloquinone Intakes According to Parenteral Nutrition Administration

Table 4 shows the total phylloquinone intake from all sources in study infants who received any period of PN and completed the study (*n* = 56). PN-derived phylloquinone intake in these 56 infants was comparable in the 0.5 mg IM (*n* = 18) and 0.2 mg IV (*n* = 21) groups, and slightly higher in the 0.2 mg IM group (*n* = 17).

Table 5 shows total phylloquinone intakes by route confined to study infants who did not receive any PN (*n* = 24).

**Table 4:** Relative intakes of phylloquinone from all sources together with total intakes at study completion in infants who received parenteral nutrition.

Phylloquinone Intake (µg)	All Infants Given PN (*n* = 56)	0.5 mg IM (*n* = 18)	0.2 mg IM (*n* = 17)	0.2 mg IV (*n* = 21)
***Via bolus doses***				
Mean (SD)	391 (277)	672 (303)	259 (94) *	275 (143) *
Median (range)	200 (200–1500)	500 (500–1500)	200 (200–400) *	200 (200–800) *
***From PN***				
Mean (SD)	1120 (940)	950 (1077)	1153 (658) ^†^	1240 (1027)
Median (range)	759 (305–5071)	589 (350–5071)	767 (515–2441) ^†^	946 (305–3895)
***Via enteral feeds***				
Mean (SD)	101 (80)	97 (63)	80 (82)	120 (89)
Median (range)	94 (2–304)	105 (2–198)	79 (6–304)	142 (8–300)
***Total phylloquinone intake µg***				
Mean (SD)	1612 (1066)	1719 (1280)	1492 (714)	1617 (1140)
Median (range)	1234 (596–6574)	1423 (956–6574)	1189 (771–2730)	1240 (596–4822)
***µg/kg***				
Mean (SD)	1168 (535)	1410 (540)	1119 (498)	1000 (506) ^‡^
Median (range)	1136 (438–2594)	1269 (721–2594)	971 (529–2057)	813 (438–2343) ^‡^
***µg/kg/day***				
Mean (SD)	37 (13)	46 (12)	34 (11) ^‡^	30 (10) *
Median (range)	33 (18–75)	44 (28–75)	33 (19–55) ^‡^	29 (18–65) *

Statistically-significant comparisons (using Mann-Whitney U test) are shown thus: * *versus* 0.5 mg IM group, *p* < 0.0001; ^†^ *Versus* 0.5 mg IM group, *p* < 0.05; ^‡^ *Versus* 0.5 mg IM group, *p* < 0.01.

**Table 5:** Relative intakes of phylloquinone from bolus doses and enteral feeds together with total intakes at study completion for infants who did not receive parenteral nutrition.

Phylloquinone Intake (µg)	All Infants Not Given PN (*n* = 24)	0.5 mg IM (*n* = 10)	0.2 mg IM (*n* = 9)	0.2 mg IV (*n* = 5)
***Via bolus doses***				
Mean (SD)	346 (202)	550 (158)	200 (NA) *	200 (NA) ^†^
Median (range)	200 (200–1000)	500 (500–1000)	200 (NA) *	200 (NA) ^†^
***Via enteral feeds***				
Mean (SD)	181 (88)	156 (107)	212 (55)	176 (94)
Median (range)	195 (7–340)	149 (7–340)	214 (132–292)	208 (14–254)
***Total phylloquinone intake µg***				
Mean (SD)	527 (187)	706 (145)	412 (55) *	376 (94) ^†^
Median (range)	466 (214–1016)	690 (507–1016)	414 (332–492) *	408 (214–454) ^†^
***µg/kg***				
Mean (SD)	322 (124)	458 (48)	220 (27) *	233 (54) ^†^
Median (range)	251 (141–562)	439 (408–562)	215 (173–249) *	249 (141-284) ^†^
***µg/kg/day***				
Mean (SD)	15 (6)	22 (3)	10 (1) *	12 (4) ^†^
Median (range)	13 (7–25)	21 (18–25)	10 (8–12) *	13 (7–17) ^†^

NA, not applicable because all infants received 0.2 mg dose. Statistically-significant comparisons (using Mann-Whitney U test) are shown thus: * *versus* 0.5 mg IM group, *p* < 0.0001; ^†^ *Versus* 0.5 mg IM group, *p* < 0.001.

#### 3.2.2. Proportional Contribution of Various Sources to Overall Phylloquinone Intakes

Table 6 shows the proportional intake of phylloquinone from each route as a percentage of the total intake, for all infants and for the subgroups. Analysis of the proportional intake of phylloquinone from the various routes shows that for the infants receiving 0.5 mg phylloquinone after delivery and who received PN, the bolus dose comprised ~40% of total phylloquinone intake over the study period, while PN comprised ~50% of intake, and enteral feeding <10% of intake. In contrast for the 0.2 mg groups, only ~20% of the total phylloquinone intake in the study period came from the bolus prophylaxis dose, whereas 70% came from PN, and <10% came from enteral feeding.

**Table 6:** Proportional intake of phylloquinone from each route from birth until study completion as a percentage of the total intake.

Proportional Phylloquinone Intake	All Infants	0.5 mg IM	0.2 mg IM	0.2 mg IV
*All Infants*	*n* = 80	*n* = 28	*n* = 26	*n* = 26
*% Via bolus doses*				
Mean (SD)	38 (23)	56 (21)	30 (16) *	27 (19) *
Median (range)	33 (5–99)	52 (23–99)	22 (7–60) *	20 (5–94) *
*% From PN*				
Mean (SD)	46 (33)	32 (26)	49 (37) ^†^	57 (31) *
Median (range)	56 (0–92)	37 (0–77)	69 (0–89) ^†^	68 (0–92) *
*% Via enteral feeds*				
Mean (SD)	17 (18)	12 (11)	21 (23)	16 (17)
Median (range)	8 (0–59)	8 (0–41)	10 (0–59)	8 (0–56)
***Infants given PN***	***n* = 56**	***n* = 18**	***n* = 17**	***n* = 21**
*% Via bolus doses*				
Mean (SD)	27 (14)	43 (11)	20 (8) *	19 (8) *
Median (range)	23 (5–64)	45 (23–64)	19 (7–42) *	17 (5–34) *
*% From PN*				
Mean (SD)	65 (16)	50 (13)	75 (10) *	71 (13) *
Median (range)	68 (28–92)	50 (28–77)	78 (54–89) *	74 (43–92) *
*% Via enteral feeds*				
Mean (SD)	8 (7)	7 (5)	6 (6)	10 (9)
Median (range)	6 (0–28)	7 (0–16)	3 (0–21)	8 (0–28)
***Infants not given PN***	**n = 24**	***n* = 10**	***n* = 9**	***n* = 5**
*% Via bolus doses*				
Mean (SD)	63 (18)	78 (13)	49 (7) *	57 (21) ^†^
Median (range)	58 (41–99)	77 (60–99)	48 (41–60) *	49 (44–94) ^†^
*% Via enteral feds*				
Mean (SD)	37 (18)	22 (13)	51 (7) *	43 (21) ^†^
Median (range)	42 (1–59)	23 (1–41)	52 (40–59) *	51 (7–56) ^†^

Statistically-significant comparisons (using Mann-Whitney U test) are shown thus: * *versus* 0.5 mg IM group, *p* < 0.0001; ^†^ *Versus* 0.5 mg IM group, *p* <0.05.

Considering infants who had not received any period of PN, the bolus dose for those receiving 0.5 mg phylloquinone after delivery represented ~80% of the total study intake (with enteral feeding ~20% of the total intake), whereas for the 0.2 mg groups the bolus dose and enteral feeding each comprised ~50% of the total phylloquinone intake.

## 4. Discussion

This is the first study to describe in detail the absolute phylloquinone intake by preterm infants from all sources during their first weeks of postnatal life. We quantified the relative proportional intake of phylloquinone from the various nutritional routes for infants who received PN and for those who did not receive PN. We found no differences in overall intakes between the three study groups that had been randomly allocated different phylloquinone regimens at birth. However after correcting for birth weight and days to study completion, the average daily intake of phylloquinone adjusted for birth weight was significantly lower for infants who had received the lower dose prophylactic bolus dose of 0.2 mg phylloquinone. We had speculated that the initial bolus doses of phylloquinone given for prophylaxis at birth would assume less importance as a source of the vitamin contributing to the overall intake at study completion, due to the increasing respective contributions to overall phylloquinone intake derived from other nutritional (dietary) sources. In infants who did not receive any PN, the bolus dose given at birth represented approximately 50% of the overall phylloquinone intake at study completion, compared to only approximately 20% in infants who had received PN.

Preterm infants represent a neglected group with respect to current knowledge of their phylloquinone intakes and recommendations for their nutritional requirements. As pointed out by Kumar *et al.* [14], published recommendations for vitamin K intakes and/or supplementation are specific for all ages except for preterm infants. As a result, recommendations for preterm infants are arbitrary and historically have ranged from 5 to 10 μg/kg/day in 1993 [15] and as high as 100 μg/kg/day in 1988 [16]. In this context it is important to note that the principles and knowledge base of dietary recommendations have changed over time. For example, the recommendations of 5–10 μg/kg/day for preterm infants in 1993 [15] came out during the lifespan of the 10th Edition of the US Food and Nutrition Board guidelines published in 1989, at which time they took the form of Recommended Daily Allowances (RDA) instead of the current Dietary Reference Intakes (DRI). At that time the RDA for term infants over the first six months was 5 μg/day, which in turn was based on the adult RDA of 1 μg/kg/day [17]. This means that the 5–10 μg/kg/day recommendation for preterm infants by Greer *et al.* [15] was 5–10 fold greater than the intake recommendations for term infants at that time. More recent guidelines published in 2005 recommended a phylloquinone intake of 8–10 μg/kg/day as an AI for preterm infants [18], representing the top end of the 1993 recommendations [15].

There is an inevitable degree of arbitrariness of recommendations even for healthy term infants. In the current United States recommendations published in 2001, the AI for healthy term infants is based on an average daily milk intake of 0.78 L and an average phylloquinone concentration in human milk of 2.5 μg/L [7]. This gave an AI of 2.0 μg/day after rounding [7]. The weak link in this calculation as a precise AI value lies with the fairly wide variations in the reported concentrations of phylloquinone in breast milk which taking the lower and upper values would result in estimated phylloquinone intakes of ~0.5 μg/day and 2.5 μg/day respectively [8].

Another weakness of current recommendations is that they are based solely on the phylloquinone content of breast milk. Although phylloquinone is the major vitamer of vitamin K in breast milk, it also contains a member of the menaquinone series, namely menaquinone-4 (MK-4) at concentrations that are about half that of phylloquinone [19,20]. There is also evidence that this MK-4 in breast milk is derived from dietary phylloquinone [20]. Future neonatal AI recommendations should also consider the contribution to intakes made by MK-4 as well as its potential contribution to neonatal vitamin K status.

Whether the dietary guidelines have been in the form of an RDA or AI, a common underlying assumption is that the infant is also given a prophylactic dose at birth in amounts recommended by the relevant paediatric societies [7,17]. For any individual infant, the optimal daily ongoing phylloquinone required from feeding will depend in part upon what prophylactic dose was given at birth and any postnatal supplementation received from PN. However there is also likely to be inter-individual variation in storage and metabolism.

For the purposes of discussion we have taken the most recent recommendation of 8–10 μg/kg/day [18] as a benchmark against which the phylloquinone intakes in this study can be compared. The median phylloquinone intake for infants who completed this study was approximately three-fold this recommended amount, and up to five to seven-fold more in some infants (Table 3). For infants who received PN, there was little difference between allocation groups. The median of the average phylloquinone intake was three to four times the recommended amount, and all had an average phylloquinone intake that ranged between ~20–70 μg/kg/day, *i.e.*, two to seven times the daily recommendation (Table 4). For infants who did not receive any PN, the median average daily intake received by 0.5 mg IM group infants was approximately twice that currently recommended, whereas for infants in the 0.2 mg IM and 0.2 mg IV bolus groups the medians and ranges of average daily intakes were remarkably close to the currently recommended intakes of 8–10 μg/kg/day (Table 5).

For infants who did not receive any PN, the bolus dose(s) of phylloquinone constituted the major source of phylloquinone intake in the study period. In contrast, for infants who had received a period of PN by far their major intake source was, somewhat surprisingly, that delivered by the PN solution. For comparison, the ratio of median phylloquinone intake from PN to that from bolus doses was ~3:1.

In infants given PN, the median overall phylloquinone intake from enteral feeds was 6% (range: 0%–28%) of the total intake. In contrast in the infants not given PN the proportional intake from enteral feeds was significantly higher, and comprised 23% of overall intake for infants receiving a 0.5 mg bolus dose of phylloquinone after delivery, and 50% of overall intake for infants who received a 0.2 mg dose. Thus the contribution towards overall phylloquinone intake made by enteral feeding is small, but becomes more important in infants not given PN and particularly so for infants who receive lower bolus prophylactic doses (0.2 mg) and who do not receive PN.

These data show that in almost all infants, the average daily phylloquinone intake was in excess of the currently recommended amounts. The intakes were particularly high at three to four times the recommended amounts in all who received PN (irrespective of prophylactic dose), and were approximately twice the recommended amounts in infants given 0.5 mg prophylactic doses who did not receive PN. Only infants who received the 0.2 mg dose and who did not receive PN had an intake which approximated well to the current recommended daily phylloquinone intake of 8–10 μg/kg/day. Our data provide further evidence that the amounts of phylloquinone currently added to manufactured PN multivitamin solutions are excessive for the needs of preterm infants and should be reviewed.

Although there are no known clinical manifestations of toxicity from phylloquinone prophylaxis in neonates, nor indeed any known toxic effects in adults consuming high amounts of vitamin K [7], we previously found evidence of hepatic overload in a sub-group of preterm infants from our main study who had received the 0.5 mg rather than the 0.2 mg dose for prophylaxis [9]. The larger dose was more likely to be associated with elevated serum phylloquinone 2,3-epoxide concentrations, and suggested overload of the hepatic vitamin K 2,3-epoxide reductase enzyme in some preterm infants [2,9]. Furthermore, in a separate study we previously obtained evidence of metabolic overload in a subgroup of preterm infants who predominately excreted the less extensively metabolised urinary 7C-side chain metabolite of vitamin K instead of the usual 5C-side chain metabolite [21]. This subgroup also had the highest serum concentrations of phylloquinone together with raised blood concentrations of phylloquinone 2,3-epoxide that is normally undetectable. This combination of increased excretion of the 7C-metabolite, high serum phylloquinone and raised phylloquinone 2,3-epoxide is indicative of a metabolic overload of both vitamin K recycling and catabolic pathways [21]. None of the infants in these studies who had biochemical evidence of overload showed any clinical manifestations of an overload status.

Because of their special nutritional requirements preterm babies are often provided with multivitamin supplements—including the fat-soluble vitamins A, D, and E—at the time of discharge home from the neonatal unit; these are usually continued throughout infancy. However post-discharge phylloquinone supplements are rarely provided to this group at present. Yet without adequate ongoing supplementation preterm infants may be at increased risk of developing vitamin K deficiency in early infancy, particularly if they continue to be exclusively breast fed. National surveillance studies of VKDB continue to report sporadic cases in preterm infants [6]. The most recent study in the United Kingdom and the Irish Republic reported a case of probable VKDB in a 24-week gestation infant who had received 0.4 mg/kg IM at birth [22]. This infant was primarily human milk fed and had no liver disease but suffered gastro-intestinal bleeding aged three months postnatal. Further study is required to assess whether phylloquinone supplements should be given routinely to preterm infants on discharge home from the neonatal unit.

## 5. Conclusions

In conclusion, this study of phylloquinone intake in the first weeks of life shows that all preterm infants received at least the minimum daily recommended intake of phylloquinone, but that most received an excessive intake. These data support the calls for the amount of phylloquinone added to standard PN multivitamin solutions to be reduced [14,23,24], and also support lower initial phylloquinone bolus doses being used for prophylaxis at birth [6]. For all infants studied, a bolus prophylactic phylloquinone dose of 0.2 mg (or ~0.3 mg/kg) was more appropriate than 0.5 mg because it resulted in an average daily intake over the study period closer to the currently recommended intake amounts. Also, average daily intakes were remarkably close to the currently recommended intake values in infants given 0.2 mg prophylaxis who did not receive any PN. In infants who do not receive PN, a 0.2 mg or 0.5 mg bolus dose given at birth represents a significant proportion of overall phylloquinone intake.

Future research in preterm infants should focus on their post-discharge vitamin K status and address the question whether additional supplementation with phylloquinone during infancy should also be routine.

## Permissions

The authors thank the American Academy of Pediatrics for kindly granting us permission to use and adapt the data presented in Table 3 from *Pediatrics* vol. 118, page e1663 [9], Copyright © 2006 by the AAP.
